# Assessment of simulated participant professional performance in health professional education: a scoping review

**DOI:** 10.1186/s41077-026-00422-1

**Published:** 2026-03-27

**Authors:** Sergio Andres León-Ariza, Elizabeth Fernanda Ríos-Villarreal, Juan Yepes-Núñez, Sebastian Castaño-Duque, Sandra X Jaramillo-Rincón

**Affiliations:** 1 School of Medicine, Universidad de Los Andes, Cra. 1 #18a-12, Bogotá, 111711 Colombia; 2 Scientific direction, Sociedad Colombiana de Anestesiología y Reanimación, Carrera 19 # 114 65, Bogotá, Colombia; 3 Anesthesia Department, Hospital Militar Central, Tv. 3C. 49 - 02, Bogotá, Colombia; 4 Innovation Office, Hospital Universitario San Ignacio, Ak 7 #40 - 62, Bogota, Colombia

**Keywords:** Scoping review, Assessment performance, Patient simulation, Education, Medical

## Abstract

**Background:**

Simulated participants (SPs) are crucial in health professional education, offering a controlled environment for students to develop clinical, diagnostic, and communication skills. However, limited research focuses on the structured evaluation of SP competencies, a key factor for maintaining educational quality. This scoping review aims to identify and categorize the strategies, tools, and instruments used to assess simulated participants’ (SPs) performance in health professional education; examine how these approaches assess and provide feedback on SP performance; and highlight gaps in the current research and practice regarding the assessment of SPs.

**Methods:**

A comprehensive literature search was conducted in Medline, EMBASE, and LILACS databases from 1990 to April 2025. Eligible studies included SP performance assessments at all levels of Miller’s pyramid (knowledge, application, demonstration, and action). Both qualitative and quantitative data were extracted and categorized based on the types of competencies assessed (communication, role accuracy, feedback) and the tools’ validation status. Descriptive statistics were employed to summarize the findings.

**Results:**

Out of 7724 articles screened, 52 studies met the inclusion criteria. Most studies (75%) did not focus exclusively on SP assessment but included it in broader educational outcomes. Commonly used assessment tools were rating scales (29%) and self-perception surveys (26%), primarily measuring role-playing accuracy and communication skills. Only 40% of these tools had undergone formal validation. Additionally, the studies often lacked consistency in the criteria used for evaluating SP performance, with a heavy focus on communication and realism. At the same time, other competencies, such as providing structured feedback and adherence to case protocols, were less frequently assessed. Tools that evaluated SP feedback delivery were found in only 21% of the studies, despite the recognized importance of this skill for effective student learning. Furthermore, several studies highlighted the need for continuous SP training and reassessment to maintain high-quality performance.

**Conclusions:**

This review identifies significant gaps in the comprehensive evaluation of SP competencies, particularly regarding the validation and standardization of assessment tools. Future research should prioritize the development of robust, validated frameworks that assess a broader range of SP skills, including feedback delivery and adherence to simulation protocols. Such advancements are essential to enhance the reliability and educational impact of SPs in health education.

**Supplementary Information:**

The online version contains supplementary material available at 10.1186/s41077-026-00422-1.

## Introduction

 Simulated participants (SPs) are defined as persons who portray a patient (Simulated participant), family member, or health care provider to meet the objectives of the simulation [1]. It refers to all human role players in any simulation context [1]. SPs are crucial in health professional education for providing a safe and controlled environment for students to practice their clinical, diagnostic, and communication skills in realistic patient encounters. SPs also offer valuable feedback on student performance, which is essential for skill development and improvement [2, 3]. However, the effectiveness of SP-based education largely depends on the SPs’ ability to deliver authentic scenarios and provide constructive feedback, alongside other contextual factors [4]. Inadequate SP performance can lead to inaccurate feedback, unrealistic practice scenarios, and a compromised educational experience for students. This can negatively impact their clinical skills and decision-making abilities, potentially affecting patient safety and outcomes [4].

Although simulated participants (SPs) are widely recognized as essential contributors to health professional education, there is no universally agreed-upon framework defining their competencies [5–7]. Prior literature has primarily concentrated on specific aspects of SP involvement, such as student perceptions of authenticity, truthfulness in role portrayal, and the delivery of feedback [8, 9]. However, broader dimensions of SP performance, including pedagogical skills, leadership, planning abilities, and thematic understanding, have been less frequently examined [5, 6, 10]. The absence of a defined competency framework may have contributed to a relative undervaluing of the systematic assessment of SPs, as compared to the emphasis placed on learners and educators.

In this context, the distinction between *simulated* and *standardized* participants becomes particularly relevant when performance assessment is considered. Standardization refers to the consistency and reproducibility of SP performance across encounters, which is essential to ensure fairness, reliability, and comparability in educational and assessment settings. From an evaluative perspective, this lack of clearly articulated and standardized competencies complicates the development of consistent, transparent, and comparable approaches to assessing SP performance across educational contexts. This underscores the need to understand how SPs’ performance has been assessed in practice and to identify gaps that hinder the development of more standardized approaches.

To frame this work, we employed two widely recognized frameworks from adjacent areas as interpretive lenses: Miller’s pyramid of clinical performance [8] and the ASPE Standards of Best Practice (SOBP) [1]. While Miller’s pyramid has traditionally been applied to the assessment of learners and the ASPE SOBP was designed to guide SP educators, both provide validated structures that can be repurposed to organize dimensions of SP performance. In this review, these frameworks were not treated as established competency models for SPs, but rather as conceptual guides that informed the categorization of findings and the development of the search strategy. Importantly, their use in this context does not imply a direct transfer of learner-centered assessment frameworks to simulated participants, but rather a pragmatic approach to structuring and interpreting heterogeneous assessment practices reported in the literature. Thus, our focus remained on how SP performance has been assessed in the literature, not on evaluating competencies that have yet to be formally defined.

Several factors influence the assessment of simulated participants. These include the diversity of SP professional backgrounds, inadequate training for SPs [9], inconsistencies in case representation, challenges related to embedded participant duties, and contextual barriers imposed by stakeholders. Such challenges highlight the need for improved training and support for SPs and coordinators and greater consistency in assessment practices. Together, these gaps justify a systematic exploration of how SP performance is currently assessed and what tools and frameworks are available to enhance its quality and reliability.

This scoping review aims to explore how simulated participants’ (SPs) performance has been assessed in health professional education. Specifically, this review aims to: (1) identify and categorize the strategies, tools, and instruments reported in the literature; (2) examine how these approaches assess and provide feedback on SP performance; and (3) highlight gaps in the current research and practice regarding the assessment of SPs. By mapping these approaches, this work seeks to provide a foundation for future efforts to enhance the quality and consistency of SP performance assessment.

## Methods

We conducted this scoping review following the Cochrane Handbook [11] for Conducting Systematic Reviews and the Joanna Briggs Institute manual [12]. We reported it using the PRISMA-ScR checklist [13].

### Research question

The research question posed for this study was adapted according to the PCCT (Population, Concept, Context, and Types of evidence) model (Table S1):

- *Population*: SP simulated/standardized patients/participants are defined as trained individuals who simulate specific medical conditions, patient behaviors, and other health system stakeholders, providing healthcare students with essential opportunities to practice and refine their clinical, diagnostic, and communication skills in a safe and controlled environment.
  In this review, we adopt the term ‘simulated participant (SP)’ per the ASPE Standards of Best Practice, which encompasses individuals portraying patients, family members, or healthcare providers. However, we recognize that much of the existing literature continues to use the term ‘simulated patient’. For this review, all references to standardized or simulated patients in the included studies were considered within the broader and updated category of ‘simulated participants. This approach ensures conceptual consistency with current best practice recommendations while acknowledging the transitional nature of the terminology in the field.
- *Concept*: strategies, tools, or instruments for assessing SP’s performance in the context of health professions teaching.
  This review distinguishes the terms ‘assessment’ and ‘evaluation’ to ensure conceptual clarity. The term ‘assessment’ describes the appraisal of specific aspects of individual SP performance, such as role portrayal, communication skills, or the quality of feedback provided. In contrast, the term ‘evaluation’ refers to broader, programmatic, or holistic approaches designed to examine SP performance, often within the context of structured training programs or institutional initiatives. This distinction was applied consistently throughout the data extraction and analysis.
- *Context*: assessment of SP performance according to Miller’s pyramid (Knows, knows how, shows how, Does) and ASPE SOBP [1, 8].
- *Types of evidence*:
  - Quantitative studies: randomized (experimental and quasi-experimental) and non-randomized (cohort studies, case-control studies, cross-sectional studies, case reports)
  - Qualitative studies: Studies that use phenomenological methods, ethnographic methods, grounded theory methods, case study models, narrative models, among others.
  - We also included evidence synthesis studies such as systematic reviews with or without meta-analysis, scoping reviews, rapid reviews, etc. as well as narrative reviews.

### Search strategy

We conducted a scoping review searching Medline, EMBASE, and LILACS databases from 1990 to April 2025. With the support of an expert librarian, we designed our search using Medical Subject Headings (MeSH) and free-text terms combined through Boolean operators. Only papers published in English and Spanish to ensure comprehensive coverage, an approach that contributes to equity by broadening the scope beyond English-dominated publications and recognizing evidence produced in diverse sociocultural and educational contexts. A detailed description of the search strategies for each database is provided in Supplementary Table S1.

Wildcard terms were intentionally used to retrieve singular and plural forms, spelling variants, and multiple lexical derivations of role-related terms, thereby maximizing the breadth and sensitivity of the search.

The protocol of this study is available in Additional file 1. It was registered in OSF with registration DOI: 10.17605/OSF.IO/Q8JMH and received approval from the institutional ethics committee number: 2023121232.

To organize and systematize the review process, we used CADIMA, a web-based tool designed for evidence synthesis screening and data extraction. The use of CADIMA underscores our commitment to a rigorous and transparent review process. CADIMA helped streamline the management of references, data extraction, and synthesis, ensuring a systematic and reliable review process.

### Selection of sources of evidence

Studies were eligible when direct or indirect information on simulated participant training or performance assessment could be extracted, even if SP evaluation was not the primary focus of the study.

To ensure a rigorous and systematic selection process, we adopted the following approach:

Two reviewers (ER and SL) independently screened the titles and abstracts of all identified articles. Any discrepancies or disagreements were resolved through consensus with a third reviewer (SJ). We standardized the full-text screening and data extraction processes to minimize potential selection bias. For this purpose, an initial reviewers’ calibration was carried out by applying the inclusion and exclusion criteria to a sample of articles previously selected by a senior researcher (JY). The random-corrected agreement for article eligibility assessment was addressed using the Kappa index. A Kappa statistic greater than 0.6 indicated substantial agreement between reviewers. Each full-text article was evaluated for eligibility based on pre-defined inclusion and exclusion criteria (ER and SL). Any discrepancies during the full-text review were resolved through consensus, with valuable input from a third reviewer (SJ) if needed, to ensure a fair and balanced decision-making process.

### Data extraction

Two independent reviewers used a standardized data extraction form to extract data (SL and ER). They discussed any disagreements and, if needed, reached consensus with a third reviewer (SJ). After extracting information based on specific research objectives (Table 1), reviewers provided a comprehensive overview of tools for assessing SP performance. Once the data extraction was completed, reviewers carried out an iterative process. SJ, ER, and SL conducted a final joint review of the extracted data and verified any missing or redundant information in the data extraction. This process led to the final list of instruments or tools for evaluating the performance of SPs in health professions teaching contexts. The following information was extracted from each manuscript: type of study population characteristics, author, year, SP training type, Validity, type of instruments.

**Table 1:** Outcomes according to research objectives

Objective	Outcome
1)Identify and characterize strategies, tools, or instruments for assessing SP competencies according to Millers’ pyramid and ASPE SOBP.	- Number and types of tools and methods identified - Classification of strategies, tools, or instruments based on their evaluative focus and population type - Description of validation status
2) Explore the role of strategies, tools, or instruments in addressing the level of performance in SP-based training.	- Level of Miller of performance - Type of performances assess
3) Highlight gaps in the current research and practice regarding assessment of SPs.	- Identification of under-researched areas - Recommendations for future research - Analysis of the alignment between existing tools and current educational needs

### Data analysis

After completing the data extraction process, reviewers carried out an iterative process to arrive at the final list of strategies, tools, or instruments for evaluating the performance of SPs in the context of health professions teaching. They also discussed and summarized in a tabular format the outcomes included in each study and the prevalence of different assessment methods.

This review uses Miller’s pyramid to categorize the competencies evaluated in SPs. Miller’s pyramid is a widely recognized framework in health professions education for assessing professional performance at different levels, making it suitable for analyzing SP competencies. To characterize the assessment instruments and tools, the research team classified the information according to the type of competency evaluated, the corresponding Miller’s level of performance, and the type of validation and scale structure.

Because it is one of the most widely recognized competency frameworks for ensuring the quality of SPs, the collected data on the type of SP performance assessed by each tool were also categorized based on the competencies outlined in the ASPE Standards of Best Practice (SOBP) [1].

This scoping review aimed to identify essential data points required for a comprehensive analysis of the included studies. In instances where specific information, such as the subjects’ competency levels based on Miller’s framework and ASPE SOBP, was not explicitly stated, we derived this information through contextual analysis of the article content. Inferences were made based on established criteria and domain knowledge to maintain consistency and analytical rigor. These data were then systematically recorded and integrated into the results. This methodological approach enabled a more thorough representation of the literature, contributing to a nuanced understanding of the competencies addressed across studies.

## Results

**Fig. 1:**
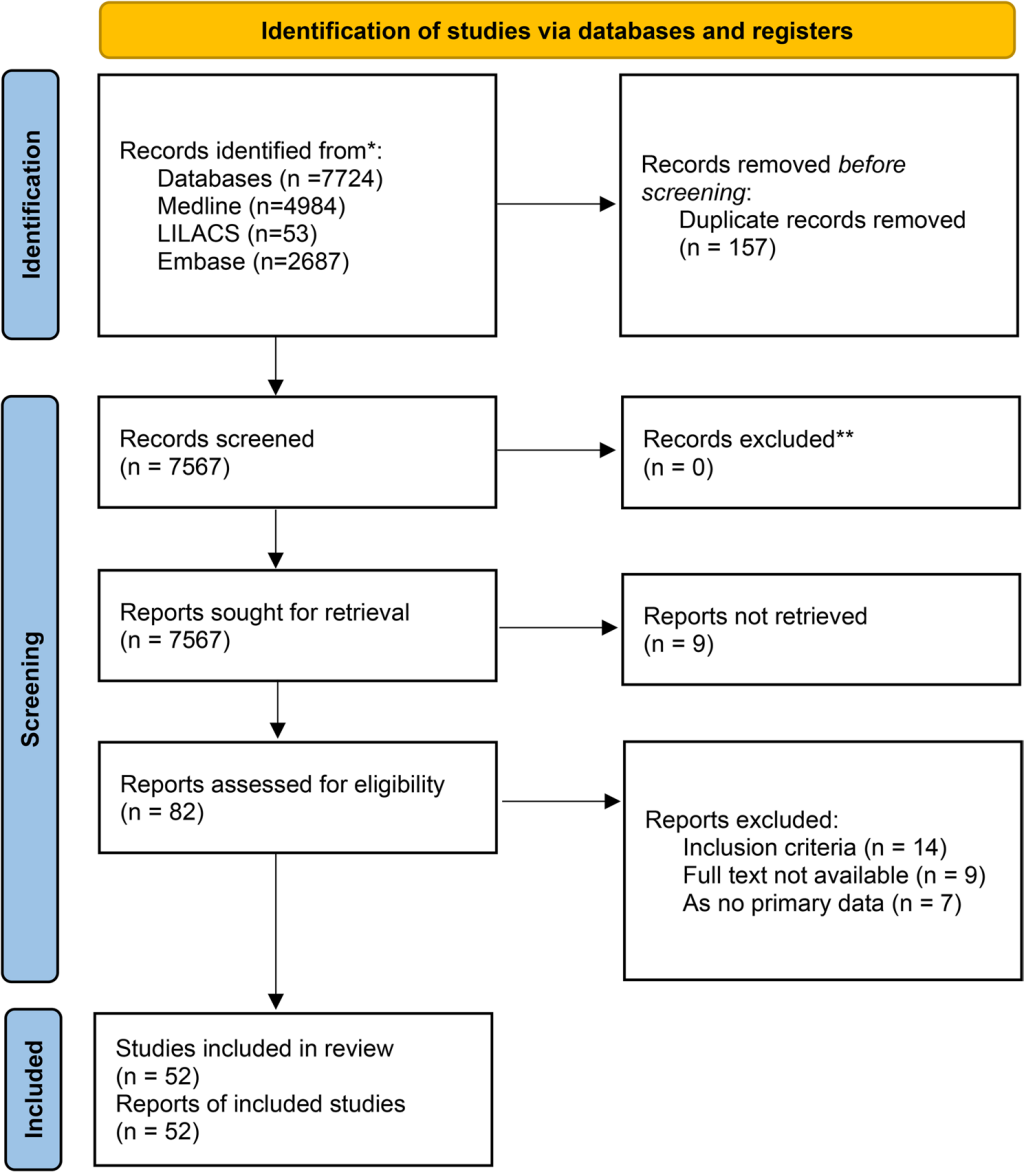
PRISMA flowchart. The PRISMA flowchart shows the number of records identified, included and excluded during the review. Adapted from Tricco et al. [13]. * PRISMA: Preferred Reporting Items for Systematic reviews and Meta-Analyses. * n: Number

Out of the 7724 records retrieved, 52 [5, 6, 10, 14–62] met our inclusion criteria. Figure 1 shows the PRISMA flowchart for the selection process. We found that 75% of the reviewed articles did not primarily focus on evaluating the performance of SP. Instead, these aspects were often addressed incidentally or as secondary considerations within the broader research methodologies employed by the studies. We interpreted these findings as indirect data. Table S2 presents the main characteristics of the studies included.

### Objective 1: strategies, tools, or instruments for assessing SP performance

The composition of our findings reflects diverse research methodologies. Table 2 visually details these proportions, and the range of methodologies employed, providing a clear overview of our review’s methodological setting.

**Table 2:** Frequency of types of studies reviewed

Type of study	*n*	%
Descriptive	28	53.8
Quasi-experimental study	8	15.4
Quantitative observational study	5	9.6
Qualitative study	3	5.8
Review	3	5.8
Mixed methods study	2	3.8
Randomized control trial	2	3.8
Systematic review	1	1.9

For the SP’s background training, we found that 78.8% (*n* = 41) received formal training [4, 6, 14–18, 20, 21, 23–27, 29–31, 34, 35, 38–52, 54, 56–58, 60–62]. This status was not reported in 15.38% (*n* = 8) of the studies [5, 19, 28, 37, 48, 53, 55, 59], and in 5.76% (*n* = 3), it was reported as the SP was untrained [32, 33, 36].

In evaluating the tools used to assess SP performance, the instruments were categorized according to their validation status and whether they employed a structured or unstructured approach (Table 3). For the purposes of this review, structured tools were defined as instruments using predefined criteria, rating scales, checklists, or rubrics, whereas unstructured tools referred to narrative comments or open-ended feedback without predefined scoring frameworks. Our findings indicate that most tools used in SP assessments are either validated and structured or unvalidated and structured, accounting for 42.3% (*n* = 22) [5, 6, 14, 15, 19, 20, 23, 24, 28, 31, 35, 36, 40, 44, 46, 50, 52, 53, 56, 57, 61, 62] and 32.69% (*n* = 17) of the instances (115,17,26,28,29,32,38,40,42,44,46–49,54,58,59), respectively. Validated but unstructured tools were used in 7.69% (*n* = 4) of the cases [16, 20, 21, 25], while unvalidated and unstructured tools were utilized in 17,3%(*n* = 9) [24, 31, 33, 36, 37, 41, 51, 55, 60].

**Table 3:** Percentage of each type of assessment validity and structure. Number and percentage of studies classified according to their validation and structuring

Type of assessment validity and structure	*n*	%
Validated/Structured	22	42.31
Unvalidated/Structured	17	32.69
Unvalidated/Unstructured	9	17,31
Validated/Unstructured	4	7.69

The assessment tools identified in the reviewed articles primarily included performance scales, perception scales, and inter-rater reliability assessments. Performance scales were used most frequently, accounting for 28.85% (*n* = 15) of the assessments [6, 13, 16, 21, 26, 28–30, 35, 38, 42, 45, 46, 48, 52], followed by perception scales at 26.92% (*n* = 14) [13, 17, 24, 27, 29, 31, 32, 34, 43, 50, 55, 56, 58, 61] and inter-rater reliability at 25% (*n* = 13) [14, 25, 28, 33, 42, 44, 45, 47, 50, 57, 59, 60, 62]. Knowledge exams [46, 48] and quantitative computerized tools [23, 53] were the least used, making up only 3.85% (*n* = 2) of the assessments. Table 4 shows a detailed breakdown of these instruments, highlighting the frequency and percentage of their usage in assessments of SP.

**Table 4:** Frequency of different types of assessment tools used in reviewed articles

Type of Tool	Number of Assessments (*n*)	Percentage (%)
Performance scale	15	28.85
Perception Scale	14	26.92
Inter-rater reliability	13	25
Feedback	11	21.15
Scores (satisfaction)	10	19.23
Station reliability, roleplay assessment	4	7.69 (each)
Other	4	7.69
Quantitative computerized tool and knowledge exams	2	3.85

### Objective 2: approaches for assessment and feedback on SP performance

ASPE outlines standards of best practices in simulation, among which is the professional training of SPs. It is important to clarify that the classification presented here was inferential, developed without a formally consensus-based framework specifically designed for assessing simulated participants. Our analysis focused not on evaluating competencies per se, but on examining performance, using validated frameworks from other areas (ASPE and Miller’s pyramid) as guiding references rather than as validation models for SP assessment. The ASPE’s Standards of Best Practice (SOBP) include, within SP training, the following components: training for completion of assessment instruments, training for role portrayal, preparation for training, reflection on the training, and feedback training.

Among the records analyzed, a total of 41 studies incorporated tools about the portrayal of standardized patient (SP) roles [5, 6, 13–17, 19–23, 26–30, 32–38, 40–46, 49–54, 56, 58, 61, 62]. Feedback training was identified in 27 instances [5, 6, 13, 15, 16, 18, 19, 21–24, 26, 31, 35, 38, 42, 44, 46–48, 50, 51, 53–55, 57, 58], while training for the completion of assessment instruments was addressed in 10 studies [13, 21, 25, 28, 39, 45, 47, 48, 59, 60]. Notably, four instruments specifically focused on training for assessment completion [47, 49, 51, 52], and an additional four studies emphasized reflection on the training process [14, 49, 52, 61].

**Fig. 2:**
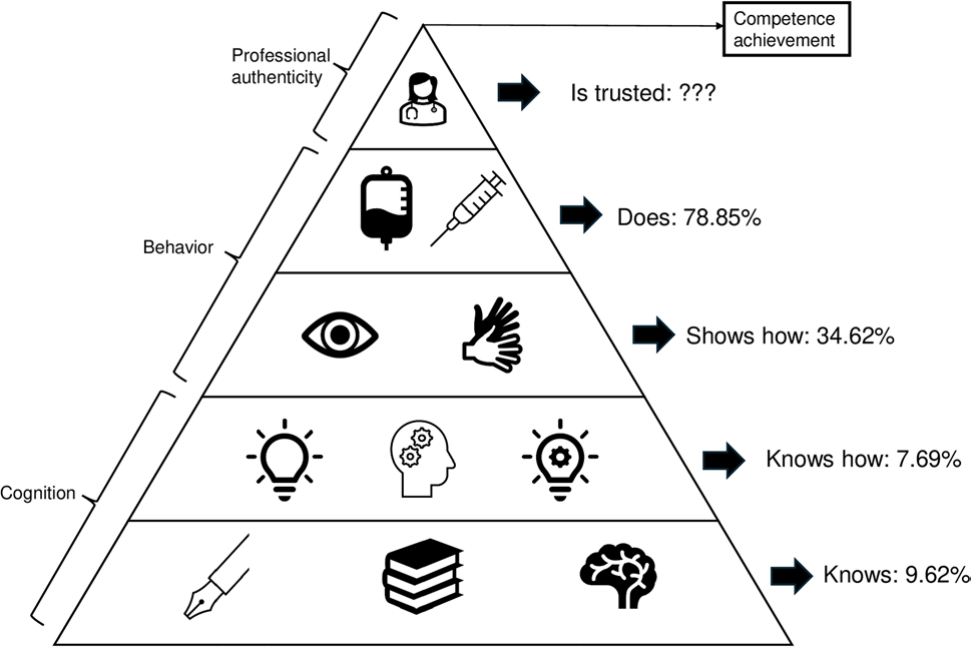
Levels of Miller’s pyramid in assessment of SP. In the assessments of the Simulated participants of the included reports, it is possible to identify the levels of Miller’s pyramid, from “Knows” to “Does”, in different proportions, reflecting the extent to which SP evaluation focuses on observable performance versus underlying knowledge or preparation. Adapted from Miller [8].

The levels of Miller’s pyramid (Fig. 2) delineate a hierarchical structure ranging from ‘Knows’ to ‘Does’. In this review, Miller’s framework was repurposed as an interpretive lens to categorize reported assessments of SP performance, rather than as a validated competency model for SPs. Within the included studies, performance was mapped across these levels in varying proportions:

- Knows: This foundational level, representing theoretical knowledge, appeared in 5 out of the total assessments [14, 23, 36, 46, 48].
- Knows how: This level evaluates the learner’s understanding of how to apply knowledge in specific contexts, appearing in 4 out of the total assessments [34, 39, 43, 60].
- Shows How: This level involves applying knowledge in a simulated or controlled environment. It was identified in 18 out of the total assessments [13, 22, 32, 34, 38, 40, 43, 45, 49, 50, 52–54, 56, 57, 60–62].
- Does: The ‘Does’ level, which represents the actual performance of tasks in a real-life or simulated setting, was the most frequently assessed, appearing in 41 out of the total assessments [5, 6, 15–31, 33–35, 37, 41, 42, 44–53, 55, 57–60, 62].

From the perspective of SP evaluation, the predominance of assessments mapped to the “Does” level suggests that most studies focused on observable SP behaviors during role portrayal and interaction, rather than on underlying knowledge or preparatory competencies. This pattern highlights an emphasis on performance-in-action, while comparatively fewer studies addressed cognitive or reflective dimensions of SP performance, such as understanding of case objectives or intentionality behind feedback delivery.

### Objective 3: gaps in research and practice on SP performance assessment

The review identified several gaps in the existing literature. Seventy-five percent of included studies did not report data on the validity and reliability of the assessment tools used for evaluating SP performance. Additionally, no studies documented the use of structured programs for comprehensively assessing SP competencies. No validated assessment tools were identified that were explicitly designed for the longitudinal monitoring of SP performance or for evaluating the relationship between training and changes in performance over time.

## Discussion

This review examined how Simulated Participant (SP) performance is evaluated in healthcare education. Our analysis of 52 studies revealed key findings with significant implications for the field.

Descriptive studies dominate current research, being the most common methodology found. It implies that the evaluated studies prioritize descriptive or observational approaches to document and analyse current practices, behaviours, or phenomena within SP practices as they naturally occur. This predominance of descriptive designs has also been observed in medical education research, where descriptive reports outweigh experimental studies [63, 64]. However, consistent with broader discussions in health professions education, recent work on SP assessment reflects an increasing interest in qualitative and mixed methods approaches, which allow for the exploration of both measurable outcomes and in-depth experiences related to SP training, performance, and pedagogical engagement [65]. Our findings align with this pattern, as qualitative and narrative methodologies were frequently used, suggesting a growing emphasis on contextual, process-oriented, and experiential dimensions of SP performance assessment.

Future research could benefit from the use of more robust experimental designs, such as randomized controlled trials, to examine the effectiveness of specific interventions. In this context, an RCT could be implemented by randomly assigning simulated participants or SP training programs to different training or assessment interventions (e.g., structured feedback training vs. standard preparation), with performance outcomes measured using standardized indicators at predefined time points.

Mixed methods designs could complement this approach by combining quantitative performance scores with qualitative data, such as SP reflections or facilitator interviews, to better understand how and why specific interventions influence performance.

Qualitative studies, in turn, remain particularly valuable for exploring contextual factors, implementation processes, and experiential dimensions of SP training and assessment that are not easily captured through standardized measures [66].

The predominance of structured tools, regardless of their validation status, suggests a clear tendency toward standardizing the observation and documentation of SP performance. However, the relatively high proportion of unvalidated structured instruments highlights an important methodological gap, as the use of predefined criteria does not necessarily ensure measurement rigor in the absence of reported validation processes. Conversely, unstructured tools, whether validated or not, were primarily used to capture contextual or qualitative aspects of SP performance and were therefore interpreted as providing exploratory rather than confirmatory evidence.

Hillier et al. [66] emphasize the importance of comprehensive SP training to ensure consistent student experiences. This aligns with our finding of a preference for structured assessment tools, particularly those aimed at promoting consistency and reliability in SP performance across encounters. While these tools offer clarity and ease of use, validated tools (whether structured or unstructured) highlight a broader need: ensuring the reliability and accuracy of assessments across simulation-based education (SBE) programs. Validated instruments such as the Maastricht Assessment of Simulated Patients (MaSP) [5, 6] provide early examples of feasible approaches, though their use remains limited.

A key aspect identified is the lack of a standardized approach for SP performance assessment. This limitation mirrors challenges in the wider simulation field, where lack of shared frameworks makes comparison across studies difficult [67]. Given the wide variability of SP uses and scenarios, this presents the challenge that an overly restrictive standardization approach could hinder their ability to adapt to the different sociocultural, educational, and healthcare contexts of the centers where they work. However, best practice standards, such as ASPE SOBP [1] or general competency frameworks, such as Miller’s pyramid [8], can serve as conceptual references for structuring assessment, even if not originally validated for SPs [68].

The distribution of SOBP-related components across the included studies suggests that SP performance is conceptualized primarily through role portrayal and feedback-related activities, with comparatively less emphasis on reflective processes and formal training for assessment completion. This pattern indicates that, in practice, SP evaluation tends to prioritize observable performance consistency over reflective or evaluative competencies. These findings highlight a potential imbalance between the technical and pedagogical dimensions of SP performance, which may limit the scope of current assessment approaches.

The Pritchard et al. [69] study offers promising insights. They found that SPs share a common understanding of what constitutes a “good” SP, identifying key attributes contributing to effective performance. This suggests the possibility of developing a more standardized SP assessment approach based on SPs’ perspectives.

Taken together, these findings further suggest the need to move beyond a focus on SP training alone and implement systematic and standardized evaluation across all aspects of SBE programs [70]. Such an emphasis may support greater consistency in educational experiences and contribute to the overall quality of simulation-based learning.

Another key finding is the emphasis on practical skills (“Does”) in the assessment of SP, aligning to provide realistic learning environments. However, the lower focus on foundational knowledge (“Knows”) could lead to gaps in theoretical learners’ understanding [71]. It is essential to acknowledge that Miller’s pyramid is hierarchical in nature, meaning that demonstration of practical skills at the “Does” level inherently implies achievement of the underlying levels (“Knows”, “Knows how”, and “Shows how”). Nonetheless, different levels may hold varying importance depending on the educational objectives. For instance, emphasizing the “Does” level aligns to create realistic learning environments that closely simulate clinical practice. This emphasis does not diminish the relevance of foundational knowledge, but reflects the priority placed on experiential learning in SBE. A balanced approach that values all levels is recommended to ensure comprehensive development while maintaining authenticity.

Our investigation also revealed a significant emphasis on acting and pedagogic skills in the assessment of SP. This focus risks overlooking other critical domains, such as planning, leadership, thematic, and emotional coping skills, which are recognized in broader competency frameworks [1, 72]. Research suggests that a variety of skills can influence SP performance but are not always considered in training, such as those mentioned previously [1, 4–6, 50, 52, 67].

While the analysis revealed a strong preference for structured SP training, a notable portion of reported practices remains undocumented or lacks formal structure. This limited reporting and heterogeneity, rather than the absence of structure per se, may contribute to inconsistencies in training outcomes and limit the ability to compare SP effectiveness across programs. Additionally, although structured assessment tools were favored, the variety of tools used highlights the need for further conceptual and methodological alignment, rather than implying deficiencies in existing practices. Taken together, these findings suggest that current research on SP assessment is fragmented and often indirect, underscoring the need for clearer reporting, dedicated frameworks, and validated tools to support interpretability and longitudinal monitoring.

Finally, current research on SP performance assessment relies heavily on indirect data, introducing potential biases. This gap has also been noted in critical reviews of simulation research [73]. Dedicated studies directly and rigorously examining SP performance are urgently needed to strengthen the evidence base and support the development of robust training and evaluation strategies.

## Conclusion

This scoping review identified significant variability in how Simulated Participant (SP) performance is evaluated in healthcare education. While the literature highlights frequent assessments at the “Does” and “Shows How” levels of Miller’s Pyramid, no consensual or clearly defined framework of competencies specific to SPs exists. Instead, a wide range of tools and approaches have been used, often focusing on discrete elements of performance rather than on comprehensive professional competencies. This lack of standardization makes it difficult to compare results across contexts and limits the ability to establish benchmarks for high-quality SP practice.

Our findings suggest that current research has focused more on assessing aspects of SP performance than on defining their competencies. This distinction is critical: performance assessments provide indirect evidence of what SPs do, but they do not by themselves constitute a competency framework. Nevertheless, the accumulated evidence from performance evaluations provides a fertile ground for beginning to conceptualize what competencies may underlie practical SP work, such as communication, role portrayal, feedback delivery, and consistency across scenarios. This review, therefore, opens the possibility of moving from fragmented performance assessment toward the exploratory development of competency frameworks tailored to SPs.

By advancing this agenda, healthcare professional education institutions could not only improve the rigor and consistency of SP assessments but also contribute to the professionalization of SP practice. Establishing exploratory competency frameworks, grounded in the evidence of what has been assessed so far, could guide future training and evaluation programs, ensuring alignment with educational goals and best practice standards. Ultimately, such progress would enhance the quality of simulation-based education and, in the long term, contribute to better-prepared health professionals and improved patient care outcomes. 

## Supplementary Information

Supplementary Material 1.

Supplementary Material 2.

Supplementary Material 3.

## Data Availability

All datasets supporting the findings of this study have been compiled and are available from the corresponding author upon reasonable request. The study protocol is publicly accessible via the Open Science Framework (OSF) at 10.17605/OSF.IO/Q8JMH. While the full data tables are not included in the manuscript or supplementary files, they can be shared for research and academic purposes upon request.
