# Supplementary material for: Assessment of simulated participant professional performance in health professional education: a scoping review
Source: Adv Simul (Lond). 2026 Mar 27;11:38. doi: 10.1186/s41077-026-00422-1 (PMC13147732; doi:10.1186/s41077-026-00422-1)
Supplement: Supplementary file 1 — Supplementary Material 1. [file 41077_2026_422_MOESM1_ESM.docx]

**Table S1. Inclusion and exclusion criteria according to PCCT model.**

|  | *Inclusion criteria* | *Exclusion criteria* |
| --- | --- | --- |
| *Population* | Individuals who fulfill the Simulated/Standardized Patients/Participants (SPs) criteria as defined previously. | Individuals involved in role-playing, including students, who act in simulations |
| *Concept* | Articles that include direct or indirect Strategies, tools and instruments for assessing SPs’ performance in education for health professions education. | Articles that include strategies, tools and instruments only for assessing student performance |
| *Context* | Articles that assess SP performance according to Miller's pyramid (Knows, knows how, shows how, Does) and ASPE Standards of Best Practices. | Articles focusing solely on using SP methodology to assess student performance and do not discuss SP performance |
| *Types of evidence* | Quantitative studies: randomized (experimental and quasi-experimental) and non-randomized (cohort studies, case-control studies, cross-sectional studies, case reports).  Qualitative studies: Studies that use phenomenological methods, ethnographic methods, grounded theory methods, case study models, narrative models, among others.  We also included evidence synthesis studies such as systematic reviews with or without meta-analysis, scoping reviews, rapid reviews, etc. as well as narrative reviews. | Any type of study that is not published in English or Spanish. |

*SP: Simulated participant*

*PCCT:* Population, Concept, Context and Types of evidence
