# Supplementary material for: Assessment of simulated participant professional performance in health professional education: a scoping review
Source: Adv Simul (Lond). 2026 Mar 27;11:38. doi: 10.1186/s41077-026-00422-1 (PMC13147732; doi:10.1186/s41077-026-00422-1)
Supplement: Supplementary file 2 — Supplementary Material 2. [file 41077_2026_422_MOESM2_ESM.docx]

**Table S2: Search strategy.**

| **Database: Medline**  **Search date: Abril 2025** |
| --- |
| 1    ("simul* patient*" or "standar* patient*" or "sample patient*" or “simu* part*” or "acto* patient*").tw.  2    ("perform* assess*" or "Skill* assess*" or "compet* assess*" or "perform* evaluat*" or "Skill* evaluat*" or "compet* evaluat*").tw.  3    ((assess* or evaluat*) adj1 (scal* or measur* or tool*)).tw.  4    (assessment or assessing).tw.  5 feedback.tw.  6 or/2-5  7 1 and 6  8    (Clinic* or hosp* or univers* or medic* or lab* or "medical center*").tw. (7629590)  9 7 and 8 |

| **Database: Embase**  **Search date: Abril 2025** |
| --- |
| #10. #9 AND [embase]/lim NOT ([embase]/lim AND    [medline]/lim)  #9.  #7 AND #8  #8.  clinic*:ab,ti,lnk,kw,tn,tt,df,mn,dn OR    hosp*:ab,ti,lnk,kw,tn,tt,df,mn,dn OR    univers*:ab,ti,lnk,kw,tn,tt,df,mn,dn OR    medic*:ab,ti,lnk,kw,tn,tt,df,mn,dn OR    lab*:ab,ti,lnk,kw,tn,tt,df,mn,dn OR 'medical    center*':ab,ti,lnk,kw,tn,tt,df,mn,dn  #7.  #1 AND #6  #6.  #2 OR #3 OR #4 OR #5  #5.  feedback:ab,ti,lnk,kw,tn,tt,df,mn,dn  #4.  assessment:ab,ti,lnk,kw,tn,tt,df,mn,dn OR    assessing:ab,ti,lnk,kw,tn,tt,df,mn,dn  #3.  ((assess* OR evaluat*) NEAR/1 (scal* OR measur*    OR tool*)):ab,ti,lnk,kw,tn,tt,df,mn,dn  #2.  'perform* assess*':ab,ti,lnk,kw,tn,tt,df,mn,dn OR    'skill* assess*':ab,ti,lnk,kw,tn,tt,df,mn,dn OR    'compet* assess*':ab,ti,lnk,kw,tn,tt,df,mn,dn OR    'perform* evaluat*':ab,ti,lnk,kw,tn,tt,df,mn,dn    OR 'skill* evaluat*':ab,ti,lnk,kw,tn,tt,df,mn,dn    OR 'compet* evaluat*':ab,ti,lnk,kw,tn,tt,df,mn,dn  #1.  'simul* patient*':ab,ti,lnk,kw,tn,tt,df,mn,dn OR    'standar* patient*':ab,ti,lnk,kw,tn,tt,df,mn,dn    OR 'sample patient*':ab,ti,lnk,kw,tn,tt,df,mn,dn    OR 'actor patient*':ab,ti,lnk,kw,tn,tt,df,mn,dn  OR ‘simulated participant’ ab,ti,lnk,kw,tn,tt,df,mn,dn |

| **Database: LILACS**  **Search date: Abril 2025** |
| --- |
| (("simul* patient*" OR "standar* patient*" OR "sample patient*" OR "actor patient*" OR “simul* part*”) ) AND (("perform* assess*" OR "Skill* assess*" OR "compet* assess*" OR "perform* evaluat*" OR "Skill* evaluat*" OR "compet* evaluat*" OR assessment OR assessing OR feedback)) |
