# Supplementary material for: Assessment of simulated participant professional performance in health professional education: a scoping review
Source: Adv Simul (Lond). 2026 Mar 27;11:38. doi: 10.1186/s41077-026-00422-1 (PMC13147732; doi:10.1186/s41077-026-00422-1)
Supplement: Supplementary file 3 — Supplementary Material 3. [file 41077_2026_422_MOESM3_ESM.docx]

**Table S3: Descriptive summary of included studies**

| **Author** | **Publication year** | **SP type** | **SP training type** | **Validity and structure** | **Strategies, tools, or instruments type** | **Type of study** | **SOBP - ASPE** | **Obtained data** |
| --- | --- | --- | --- | --- | --- | --- | --- | --- |
| Abdelkhalek, Nahed M.; Hussein, Amal M.; Sulaiman, Nabil; Hamdy, Hossam | 2009 | Formal professional education | Formal training | Validated/structured | Validated/structured | Quasi-experimental study | Training for role portrayal | Assessment of the performance and feedback of the SPs by students and teachers. |
| Aso, Ryoko; Inoue, Chikako; Yoshimura, Akinobu; Shimura, Toshiro | 2013 | Volunteers | Formal training | Validated/structured | Validated/structured | Descriptive | Training for role portrayal, Training for feedback | Assessment of the knowledge to answer and improvise the questions correctly |
| Austin, Zubin; Gregory, Paul; Tabak, Diana | 2006 | Formal professional education | Formal training | Unvalidated/structured | Unvalidated/structured | Descriptive | Training for role portrayal | Assessment of student satisfaction in relation to the performance of SP |
| Baig, Lubna A.; Beran, Tanya N.; Vallevand, Andrea; Baig, Zarrukh A.; Monroy-Cuadros, Mauricio | 2014 | Formal professional education | Formal training | Validated/unstructured | Validated/unstructured | Descriptive | Training for role portrayal | The accuracy of the SPs was asessed by measuring interobserver correlation and applying performance scales to videos from multiple OSCE stations. |
| Black, Stephen A.; Nestel, Debra F.; Horrocks, Emma J.; Harrison, Rachael H.; Jones, Norma; Wetzel, Cordula M.; Wolfe, John H. N.; Kneebone, Roger L.; Darzi, Ara W. | 2006 | Formal professional education | Formal training | Unvalidated/structured | Unvalidated/structured | Descriptive | Training for role portrayal | Assessment of the perception of ratings of realism of the use of SP in hybrid surgical scenarios |
| Bokken, Lonneke; Linssen, Tim; Scherpbier, Albert; van der Vleuten, Cees; Rethans, Jan-Joost | 2009 | Both | Undetermined | Validated/structured | Validated/structured | Quasi-experimental study | Training for role portrayal, Training for feedback | Assess of the capacity and extent of feedback administration by the SP from the patient’s perspective in the domains of clinical skills and communication skills. |
| Bokken, Lonneke; Rethans, Jan-Joost; Jobsis, Quirijn; Duvivier, Robbert; Scherpbier, Albert; van der Vleuten, Cees | 2010 | Both | Formal training | Validated/structured | Validated/structured | Descriptive | Training for role portrayal, Training for feedback | Performance was compared between SPs and real patients using satisfaction scores. |
| Bouter, Shifra; van Weel-Baumgarten, Evelyn; Bolhuis, Sanneke | 2013 | Formal professional education | Formal training | Validated/structured | Validated/structured | Qualitative | Training for role portrayal | Validation of the NESP scale as a feasible, valid, and reliable instrument that could be used to assess the performance of individual SPs. |
| Cho, Jonathan C.; Wallace, Takova D.; Yu, Frank S. | 2019 | Formal professional education | Formal training | Validated/unstructured | Validated/unstructured | Descriptive | Training for role portrayal | Performance was compared between SPs and students as patients using satisfaction scores. |
| Chuisano, Samantha A.; Anderson, Olivia S.; Weirauch, Katrina; Roper, Rosemary; Phillips, Julie; McCabe, Carolyn; Sadovnikova, Anna | 2022 | Formal professional education | Formal training | Validated/unstructured | Validated/unstructured | Descriptive | Training for role portrayal, training for feedback, | Assessment of SP performance as part of the evaluation of an educational strategy. |
| Collins, J.P.; Harden, R.M. | 1998 | Volunteers | Formal training | Validated/structured | Validated/structured | Descriptive | Training for role portrayal, training for feedback, | Formative assessment within a Feedback training program for PS with co-evaluation in roleplay strategy and feedback by coaches. |
| Coro-Montanet G, Pardo Monedero MJ, Sánchez Ituarte J, Wagner Porto Rocha H, Gomar Sancho C | 2023 | Formal professional education | Formal training | Validated/structured | Validated/structured | Descriptive | Training for role portrayal | Assessment of the capacity of SP as co-evaluator in interprofessional collaboration through Interrater Reliability of Standardized Actors Versus Non actors |
| Dayer Berenson, Linda; Goodill, Sharon W.; Wenger, Sarah | 2012 | Undetermined | Formal training | Unvalidated/unstructured | Unvalidated/unstructured | Randomized Controlled Trial | Training for role portrayal, training for feedback, | Explore pharmacy students’ perspective through perception scales on the performance of the PS during the feedback |
| Dickter, David N.; Stielstra, Sorrel; Lineberry, Matthew | 2015 | Formal professional education | Formal training | Validated/unstructured | Validated/unstructured | Descriptive | Preparation for training, Training for role portrayal, Training for feedback | Compare the scores of examiners and simulated participants in the assessment of communication skills in palliative medicine |
| Doyle AJ, Sullivan C, O'Toole M, Tjin A, Simiceva A, Collins N, Murphy P, Anderson MJ, Mulhall C, Condron C, Nestel D, MacAulay R, McNaughton N, Coffey F, Eppich W | 2024 | Both | Formal training | Structurated/unvalidated | Structurated/unvalidated | Review | Training for role portrayal, training for feedback | The PS's performance during training for a clinical communication skills course is evaluated using scales from a course on perception scales. |
| ElGeed, Hager; El Hajj, Maguy Saffouh; Ali, Raja; Awaisu, Ahmed | 2021 | Formal professional education | Undetermined | Validated/structured | Validated/structured | Descriptive | Training for feedback | The study assessed authenticity of SPs using MaSP rubric in substance abuse treatment scenarios |
| Finlay, I. G.; Stott, N. C.; Kinnersley, P. | 1995 | Volunteers | Formal training | Unvalidated/structured | Unvalidated/structured | Descriptive | Training for completion of assessment instruments, Training for role portrayal | The use of formative observation is suggested within the training processes to administer effective feedback. |
| Franco, Camila; Franco, Renato; Severo, Milton; Ferreira, Maria Amelia | 2016 | Formal professional education | Formal training | Unvalidated/structured | Unvalidated/structured | Descriptive | Training for role portrayal, training for feedback | It was compared by means of perception scales on realism of the scenario to SP versus virtual patients. |
| Fussell, Holly E.; Lewy, Colleen S.; McFarland, Bentson H. | 2009 | Formal professional education | Formal training | Validated/structured | Validated/structured | Descriptive | Training for role portrayal, training for feedback, | Assessment of realistic performance and accuracy in completing the checklist, as part of SP training strategies in measure quality programs. |
| George, Riya Elizabeth; Wells, Harvey; Cushing, Annie | 2022 | Formal professional education | Formal training | Unvalidated/structured | Unvalidated/structured | Quasi-experimental study | Preparation for training, Training for role portrayal, Reflection on the training process | Validation of a standardized patient performance rating scale - student version |
| Gesundheit, Neil; Brutlag, Pauline; Youngblood, Patricia; Gunning, William T.; Zary, Nabil; Fors, Uno | 2009 | Formal professional education | Untrained | Unvalidated/unstructured | Unvalidated/unstructured | Descriptive | Training for role portrayal, training for feedback | No formal data is described about the assessment of PS performance . Importance of observation of SP during the training process. |
| Glassman, P. A.; Luck, J.; O'Gara, E. M.; Peabody, J. W. | 2000 | Formal professional education | Untrained | Unvalidated/structured | Unvalidated/structured | Quasi-experimental study | Training for completion of assessment instruments, training for role portrayal | Assessment of the performance and feedback of the EPs by students' and teachers' perceptions in a teaching strategy in psychiatry. * |
| Godzik CM, Solomon J, Yacinthus B | 2022 | Formal professional education | Formal training | Unvalidated/unstructured | Unvalidated/unstructured | Descriptive | Training for role portrayal | No formal data is described in relation to the assessment of PS performance but use feedback in the training process. |
| Gonullu, Ipek; Dogan, Celal Deha; Erden, Sengul; Gokmen, Derya | 2023 | Formal professional education | Formal training | Validated/structured | Validated/structured | Descriptive | Training for role portrayal, Training for feedback | Assessment of effectiveness of Patient Instructors (PIs) compared to teaching staff in providing feedback to medical students. through a 12-item questionnaire |
| Gormley, Gerry; Sterling, Margaret; Menary, Allison; McKeown, Gary | 2012 | Formal professional education | Untrained | Validated/structured | Validated/structured | Descriptive | Training for feedback, training for role portrayal | Assess how effectively of SPs to conduct consistent and reliable assessments of nursing students' communication practices, using interrater reliability agreement indices. |
| Herrmann-Werner A, Erschens R, Fries M, Wehner H, Zipfel S, Festl-Wietek T | 2022 | Formal professional education | Undetermined | Unvalidated/unstructured | Unvalidated/unstructured | Descriptive | Training for completion of assessment instruments | Presents a teaching and training strategy for Standardized Patients (SPs). It outlines various evaluation milestones, including the use of multiple-choice exams, video feedback, and performance checklists and calculating reliability scores for SPs. |
| Himmelbauer, Monika; Seitz, Tamara; Seidman, Charles; Loffler-Stastka, Henriette | 2018 | Formal professional education | Formal training | Unstructured/unvalidated | Unstructured/unvalidated | Quasi-experimental study | Training for completion of assessment instruments | The ability of the SP to be an observer and evaluator during the OSCE stations is evaluated in comparison with clinical teachers. |
| Huang M, Yang H, Guo J, Fu X, Chen W, Li B, Zhou S, Xia T, Peng S, Wen L, Ma X, Zhang Y, Zeng J | 2024 | Formal professional education | Formal training | Unvalidated/structured | Unvalidated/structured | Review | Training for role portrayal | Talk about the limitation of Likert scales in the assessment of SP performance because it can limit observable improvement measurement due to the instrument's constraints. Additionally, suggest the importance of noting that giving constructive observation. |
| Iñiguez RX, Figueroa Narváez JA, Diamond LC, Gregorich SE, Karliner L, González J, Pérez-Cordón C, Shin TM, Izquierdo K, Ortega P | 2024 | Formal professional education | Formal training | Validated/structured | Validated/structured | Descriptive | Training for feedback, training for role portrayal | This article does not describe the evaluation process per se; however, it shows how SPs expect to be assessed and given feedback within their roles. * |
| Kissela, Brett; Harris, Steven; Kleindorfer, Dawn; Lindsell, Christopher; Pascuzzi, Robert; Woo, Daniel; Szaflarski, Jerzy; Kanter, Daniel; Schneider, Alex; Sostok, Michael; Broderick, Joseph | 2006 | Formal professional education | Formal training | Unvalidated/stuctured | Unvalidated/stuctured | Descriptive | Training for role portrayal, training for feedback, Training for completion of assessment instruments | Focuses on self-assessment and co-assessment of SPs' performance as part of an SP training program. |
| Krautter, Markus; Diefenbacher, Katja; Schultz, Jobst-Hendrik; Maatouk, Imad; Herrmann-Werner, Anne; Koehl-Hackert, Nadja; Herzog, Wolfgang; Nikendei, Christoph | 2017 | Volunteers | Formal training | Unvalidated/unstructured | Unvalidated/unstructured | Systematic review | Training for feedback | Perception assessment of simulated participants' feedback on the acquisition of communication skills. * |
| Luebbert R, Perez A, Andrews A, Webster-Cooley T | 2023 | Undetermined | Formal training | Unvalidated/structured | Unvalidated/structured | Descriptive | Training for feedback | Perception assessment of simulated participants' feedback on optometry students. |
| MacLean, Sharon; Geddes, Fiona; Kelly, Michelle; Della, Phillip | 2018 | Formal professional education | Formal training | Validated/structured | Validated/structured | Descriptive | Training for feedback | Assessed of residents' perception of the credibility of SP versus faculty feedback |
| Mafinejad, M.K.; Rastegarpanah, M.; Moosavi, F.; Shirazi, M. | 2017 | Volunteers | Formal training | Unvalidated/structured | Unvalidated/structured | Descriptive | Training for feedback | Assessed of residents' perception of the credibility of SP versus dentists faculty assessment skills |
| May, Win | 2008 | Formal professional education | Formal training | Validated/structured | Validated/structured | Quasi-experimental study | Training for feedback, training for role portrayal | Validation of the Maastricht Assessment of Simulated participants (MaSP)  like a valid, reliable, and feasible instrument for evaluating the individual performance of SPs. |
| McLaughlin, Kevin; Gregor, Laura; Jones, Allan; Coderre, Sylvain | 2006 | Formal professional education | Formal training | Unvalidated/structured | Unvalidated/structured | Descriptive | Training, for role portrayal, Training for completion of assessment instruments, Training for feedback | Assessment skill of SP versus dentists faculty |
| Nelson, Gavin | 2022 | Undetermined | Undetermined | Unvalidated/structured | Unvalidated/structured | Descriptive | Training for completion of assessment instruments, Training for feedback | Use of the MaSP instrument to assess performance of SP on pharmacy education |
| Nestel, Debra; Clark, Susan; Tabak, Diana; Ashwell, Victoria; Muir, Elizabeth; Paraskevas, Paraskeva; Higham, Jenny | 2010 | Formal professional education | Formal training | Unvalidated/structured | Unvalidated/structured | Descriptive | Preparation for training, Training for completion of assessment instruments, Training for feedback | SPs accuracy, consistency, replicability, and portability should be evaluated |
| Perera, Jennifer; Perera, Joachim; Abdullah, Juriah; Lee, Nagarajah | 2009 | Formal professional education | Formal training | Validated/structured | Validated/structured | Qualitative | Preparation for training, reflection on the training process, training for role portrayal | Compares the trained student peers versus paid actors as standardized patients for counseling |
| Qureshi, Ayesha Aleem; Zehra, Tabassum | 2020 | Volunteers | Formal training | Unvalidated/unstructured | Unvalidated/unstructured | Descriptive | Training for role portrayal, reflection on the training process | Evaluated SP assessment skills on communication and counseling of pharmacy students |
| Resende, K.A.; Cavaco, A.M.; Luna-Leite, M.A.; Acacio, B.R.; Pinto, N.N.; Neta, M.D.S.; Melo, A.C. | 2020 | Volunteers | Formal training | Validated/structured | Validated/structured | Qualitative | Training for role portrayal | Evaluated the training and standardization methods of multiple (SPs) performing a single scenario in a multicenter study |
| Rivière E, Der Sahakian G, Tremblay ML, Chiniara G; QASSH working group | 2025 | Undetermined | Undetermined | Validated/structured | Validated/structured | Mixed methods study | Training for role portrayal, Reflection on the training process | Provided validity evidence for QASSH tool to assess the quality of healthcare simulation scenarios. |
| Shah, Rakhee; Ctori, Irene; Edgar, David F.; Parker, Pam | 2021 | Formal professional education | Formal training | Unvalidated/structured | Unvalidated/structured | Observational quantitative | Training for completion of assessment instruments | Comparison of evaluation with a checklist between SP and VCTs. |
| Stroud, Lynfa; Sibbald, Matthew; Richardson, Denyse; McDonald-Blumer, Heather; Cavalcanti, Rodrigo B. | 2018 | Undetermined | Undetermined | Unvalidated/unstructured | Unvalidated/unstructured | Quasi-experimental study | Training for role portrayal | Examined resident perception of feedback provided by faculty vs SP during a formative OSCE. |
| Tran NN, Thomas D, Haverkamp CF, Leslie EL, Kashmer D | 2023 | Volunteers | Formal training | Validated/structured | Validated/structured | Quantitative observational | Training for role portrayal | Addressed the student’s satisfaction with their mock SP experience. |
| Williams, Brent C.; Hall, Karen E.; Supiano, Mark A.; Fitzgerald, James T.; Halter, Jeffrey B. | 2006 | Volunteers | Formal training | Validated/structured | Validated/structured | Mixed methods study | Training for role portrayal | Utility and feasibility of the GSPI |
| Willson, M.N.; McKeirnan, K.C.; Yabusaki, A.; Buchman, C.R. | 2021 | Both | Formal training | Unvalidated/structured | Unvalidated/structured | Observational quantitative | Training for role portrayal, training for feedback | Compared ACSPs and PASPs based on student preference for SP type, and the SPs’ acting performance. |
| Wind, Lidewij A.; Van Dalen, Jan; Muijtjens, Arno M. M.; Rethans, Jan-Joost | 2004 | Both | Undetermined | Validated/structured | Validated/structured | Observational quantitative | Training for role portrayal | Validation of the Maastricht Assessment of Simulated participants (MaSP)  like a valid, reliable, and feasible instrument for evaluating the individual performance of SPs. |
| Wiskin, C. M.; Elley, K.; Jones, E.; Duffy, J. | 2013 | Undetermined | Undetermined | Unvalidated/structured | Unvalidated/structured | Prospective, randomized controlled trial | Training for completion of assessment instruments | Assessment skill of SP versus dentists faculty |
| Wollney EN, Vasquez TS, Stalvey C, Close J, Markham MJ, Meyer LE, Cooper LA, Bylund CL | 2023 | Formal professional education | Formal training | Unvalidated/unstructured | Unvalidated/unstructured | Quantitative quasi-experimental | Training for role portrayal, training for feedback | Examined the differences between ratings of SPs and a set of outside observers with training OSCEs. |
| Wu ML, Chao LF, Hung YT | 2024 | Volunteers | Formal training | Validated/structured | Validated/structured | Literature review | Training for role portrayal, training for feedback | Evaluated examinees’ perceptions of the performance of student standardized patients (SPs). |
| Yao F, Chen JS, Chen LH, Ye BY, Yang CJ, Li XZ | 2025 | Undetermined | Formal training | Validated/structured | Validated/structured | Longitudinal study | Training for role portrayal | Evaluated satisfaction with the application of SP in a certification assessment for residents. |
